# Therapies for obsessive-compulsive disorder: Current state of the art and perspectives for approaching treatment-resistant patients

**DOI:** 10.3389/fpsyt.2023.1065812

**Published:** 2023-02-16

**Authors:** Kevin Swierkosz-Lenart, Joao Flores Alves Dos Santos, Julien Elowe, Anne-Hélène Clair, Julien F. Bally, Françoise Riquier, Jocelyne Bloch, Bogdan Draganski, Marie-Thérèse Clerc, Beatriz Pozuelo Moyano, Armin von Gunten, Luc Mallet

**Affiliations:** ^1^Department of Psychiatry, Service Universitaire de Psychiatrie de l’Age Avancé (SUPAA), Centre Hospitalier Universitaire Vaudois, Prilly, Switzerland; ^2^Department of Mental Health and Psychiatry, Geneva University Hospital, Geneva, Switzerland; ^3^Department of Psychiatry, Lausanne University Hospital, University of Lausanne, West Sector, Prangins, Switzerland; ^4^Department of Psychiatry, Lausanne University Hospital, University of Lausanne, North Sector, Yverdon-les-Bains, Switzerland; ^5^Sorbonne University, UPMC Paris 06 University, INSERM, CNRS, Institut du Cerveau et de la Moelle Épinière, Paris, France; ^6^Department of Clinical Neurosciences, Service of Neurology, Lausanne University Hospital and University of Lausanne, Lausanne, Switzerland; ^7^Department of Clinical Neuroscience, Service of Neurosurgery, Lausanne University Hospital (CHUV), University of Lausanne (UNIL), Lausanne, Switzerland; ^8^Laboratory for Research in Neuroimaging (LREN), Department of Clinical Neurosciences, Centre for Research in Neurosciences, Lausanne University Hospital, University of Lausanne, Lausanne, Switzerland; ^9^Department of Neurology, Max Planck Institute for Human Cognitive and Brain Sciences, Leipzig, Germany; ^10^Univ Paris-Est Créteil, DMU IMPACT, Département Médical-Universitaire de Psychiatrie et d’Addictologie, Hôpitaux Universitaires Henri Mondor - Albert Chenevier, Assistance Publique-Hôpitaux de Paris, Créteil, France; ^11^Sorbonne Université, Institut du Cerveau - Paris Brain Institute - ICM, Inserm, CNRS, Paris, France

**Keywords:** obsessive-compulsive disorder, deep brain stimulation, neuromodulation, resistant, refractory

## Abstract

Even though obsessive compulsive disorder (OCD) is one of the ten most disabling diseases according to the WHO, only 30–40% of patients suffering from OCD seek specialized treatment. The currently available psychotherapeutic and pharmacological approaches, when properly applied, prove ineffective in about 10% of cases. The use of neuromodulation techniques, especially Deep Brain Stimulation, is highly promising for these clinical pictures and knowledge in this domain is constantly evolving. The aim of this paper is to provide a summary of the current knowledge about OCD treatment, while also discussing the more recent proposals for defining resistance.

## 1. Introduction

According to the DSM-5, Obsessive-Compulsive Disorder (OCD) is characterized by the presence of obsessions and/or compulsions. Obsessions are recurrent and persistent thoughts, urges, or images that are experienced as intrusive and unwanted, whereas compulsions are repetitive behaviors or mental acts that an individual feels driven to perform in response to an obsession or according to rules that must be applied rigidly (1).

The WHO listed OCD within the ten medical illnesses associated with greatest worldwide disability (2), its estimated prevalence in the United States is 2.3% for lifetime OCD and 1.2% for 12 months criteria (3), while the lifetime prevalence of OCD in the general population, according to a study that considered six European countries, is estimated to be in the range of 1–2% (4). Despite the major impact of this condition on quality of life, it has been reported that only a small proportion of OCD sufferers seek psychiatric treatment, ranging from 30 to 40% (5).

Patient reluctance to consult a professional, together with the fact that OCD rarely results in situations requiring compulsory hospitalization, probably accounts for psychiatrists’ lack of opportunity to recognize and treat this condition, as found in several surveys (6, 7).

These critical issues constitute a potential risk that many patients do not access adequate treatment and will be misdiagnosed as resistant when several available treatment steps have not been offered.

The aim of this paper is to provide a summary of the current knowledge about OCD treatment, while also discussing the more recent proposals for defining resistance.

## 2. First-line treatment

### 2.1. Psychotherapy

Cognitive Behavioral Therapy (CBT) with exposure and response prevention (E/RP) is one of the first-line evidence-based treatments for OCD (8, 9). Indeed, several meta-analyses have found a significant reduction of OCD symptoms after a psychotherapy including E/RP (10–13), with 42–52% of patients achieving symptom remission (12). Moreover, CBT has been found to be more efficient than serotoninergic treatment, including Selective Serotonin Reuptake Inhibitors (SSRIs), by several studies (12, 14, 15). More recently, a review indicated a number needed to treat (NNT) of three for CBT and five for SSRIs (16), with the additional benefit of fewer side effects and relapses. However, those results should be interpreted considering potential biases, such as the exclusion from the CBT trials of patients with comorbidities or the most severe cases of OCD.

Furthermore, the limit of accessibility to CBT should be considered, SSRI remaining the most cost-effective treatments (17). Indeed, financial cost, difficulty attending sessions and fear regarding anxiety-provoking exercises are the main perceived barriers to initiate and complete CBT (18). In line with those results, a systematic meta-analysis indicated that more than 15% of eligible patients refuse CBT and about 16% dropped out, with lower dropout rates in group CBT (19), Internet based CBT (20) or other psychotherapy techniques combined with E/RP, such as acceptance and commitment therapy (ACT) could partially overcome those limits.

Nevertheless, it is suggestive of the crucial importance of psychotherapy in the treatment algorithm, especially since acceptance and commitment therapy (ACT) (21) or mindfulness (22), alone or combined with E/RP, showed promising perspectives in the treatment of OCD.

### 2.2. Pharmacotherapy: Serotoninergic agents

Along with CBT, SSRIs are considered a first-line treatment in OCD by the American Psychiatric Association (APA) Practice Guidelines (8). There is multiple evidence regarding the connections between serotonergic disruption and OCD: a specific genetic polymorphism for the gene encoding the serotonin transporter 5-HTT (SLC6A4) has been found in significant association with OCD patients (23, 24). An increased sensitivity of 5-HT2 receptors has also been hypothesized in OCD patients, since OCD patients show a more pronounced neuroendocrine response than healthy controls to stimulation with an agent with high affinity for 5-HT2 receptors (25). These neurophysiological findings may explain the low efficacy of antidepressants with primary norepinephrine action, such as desipramine, compared with molecules with a serotonergic action profile (26). However, there is no evidence to date of an unequivocal correlation between alterations in specific serotoninergic pathways and the clinical manifestation of symptoms. As noted in a recent comprehensive review of pharmacotherapeutic strategies, considerations of serotonergic disruption are based exclusively on empirical evidence, whereas studies on specific alterations of 5HT2A receptors have produced controversial results (27).

As such, SSRI monotherapy is suggested as an option for patients with insufficient compliance for psychotherapy. Clomipramine has been accounted for a greater efficacy in several meta-analyses (28, 29), but single trials (30, 31) comparing it head-to-head with SSRIs do not support this evidence. When SSRIs are used to treat OCD, they should be regarded as anti-obsessive agents rather than antidepressants, bearing in mind that both the dosage and the latency between the start of treatment and the response are different when compared to depression. For example, SSRI are more efficacious when used at higher doses than for depression (32, 33). Also, a meta-analysis showed that the minimum time between SSRI initiation at effective dosage and its clinical impact is 10–12 weeks (32). However, this work, which reviews seventeen randomized clinical trials, introduces an element of complexity. The first statistically significant results of symptom reduction are observed after only 2 weeks of prescribing SSRIs, and the improvement follows a logarithmic curve whereby the greatest effects of treatment are observed in the early phase. Some meta-analyses even suggest longer waiting periods, showing a progressive improvement up to 28 weeks after the initiation of SSRI therapy (34–36).

However, the framework of action of serotonergic agents remains complex and not unambiguously definable in terms of both dosage and response delay. A meta-analysis focusing on the question of correlation between dosage and clinical response, conducted on nine randomized clinical trials, concluded that there was a 7–9% higher reduction in OCD symptoms in patients in the high-dose group, although all treatment groups (low-dose, medium-dose, and high-dose) showed a greater reduction in YBOCS score than placebo group (37). Although the benefit of these higher doses has been shown, it must be born in mind that the number needed to treat (NTT) of OCD patients on monotherapy with standard-dose SSRIs is five, whereas the NTT for obtaining a response by switching to a medium or high dose ranges from 13 to 15. This testifies to the limited possibility of obtaining a response in a non-responder with dose escalation (33). A further factor to be taken into account is the fact that off-label prescriptions produce a considerable lack of access for patients to adequate dose therapy. This phenomenon prolongs the duration of untreated illness (DUI), a parameter that appears to have a significant impact in terms of outcome for patients treated with SSRIs (38).

Table 1 indicates the maximum dosage for several SSRIs used as anti-obsessive agents (26), compared to the same molecules when used as antidepressant agents.

**TABLE 1:** Comparison of maximum dosage of selective serotonin re-uptake inhibitors (SSRIs) when used as antidepressants vs. anti-obsessive agents.

Serotonine reuptake inhibitors	Maximum dosage as antidepressant	Maximum dosage as anti-obsessive	Occasionally prescribed (e.g., rapid metabolizers)
Escitalopram	20 mg	40 mg	60 mg
Fluoxetine	60–80 mg	80 mg	120 mg
Fluvoxamine	300 mg	300 mg	450 mg
Paroxetine	40 mg	60 mg	100 mg
Sertraline	200 mg	200 mg	400 mg

The use of high doses of serotonergic agents requires subsequent medical monitoring, in particular ECG monitoring (risk of QT prolongation), liver enzymes and electrolytes check at month 1, 2, 3, 6, and 12 after treatment initiation, then once a year in the absence of side effects. If necessary, drug plasma level controls and CYP450 genotyping may be useful in case of suspicion of rapid metabolizers. Particular attention must be paid when other drugs are prescribed for co-morbid conditions, to prevent serotoninergic syndrome (for instance monoamine oxidase B inhibitors).

The main psychiatric assessment tool in the follow-up of OCD therapy is the YBOCS. Data from the literature define a favorable response in terms of a reduction from the initial score of 25–35%, and there is not a generally accepted consensus in defining this threshold (39). The International Treatment Refractory OCD Consortium proposed stages for assessing the response to treatment. A reduction of 35% or greater of the YBOCS score and Clinical Global Impression (CGI) less or equal to two is considered a full response; a reduction between 25 and 35% is a partial response and a reduction inferior to 25% is a non-response (40). A retrospective study of 87 adult patients attempted to establish a correlation between the percentage reduction of YBOCS and CGI. The results show that setting a 30% or greater reduction in YBOCS has the highest efficiency of clinical predictivity, with a 91% chance of having a CGI corresponding to “improved” or “very much improved” (41).

## 3. Second-line treatment

Taking for granted the heterogeneity in the definition of response criteria in the literature, a recent review on pharmacotherapeutic strategies in OCD suggests that only up to 50% of patients respond to SSRIs (27). Despite research aimed at identifying the preferred molecule among SSRIs, no significant differences in terms of efficacy have been shown within this class (39, 42). Switching from one SSRI to another seems to allow an improvement of 20% in the best cases. Alternative strategies are discussed separately in the following sections.

### 3.1. Clomipramine

Clomipramine is a tricyclic antidepressant. Its antidepressant properties are probably due to the inhibition of neuronal re-uptake of serotonin (5-HT) and noradrenaline released into the synaptic space. Clomipramine’s pharmacological spectrum includes noradrenergic, antihistaminic and serotonergic properties. Its role in the treatment of OCD has been established since the first controlled study in 1991 (43), and its effectiveness has been confirmed several times in subsequent studies (15, 44). Despite rapidly gaining a reputation as the gold standard treatment for OCD, clomipramine showed to be non-superior to SSRIs in a recent meta-analysis including 53 articles (42). Its side-effect profile (including epilepsy, increased liver enzymes, xerostomia, increased heart rate, constipation) calls for caution when prescribing it.

Although clomipramine remains a possible second-line treatment according to the APA (8) and Canadian clinical practice guidelines (45), the most recent evidence suggests that switching from an SSRI to clomipramine is not mandatory, while preliminary data support its use as an add-on agent in cases of resistance. Further investigations in this regard remain necessary (42).

### 3.2. SNRIs

Venlafaxine is the most studied molecule in this class, having shown efficacy in numerous trials (45–47) at a dose of 150–375 mg/d, with a response rate of up to 60%. The interpretation of these data is limited by the heterogeneity of responder definition: 35% reduction in YBOCS (46); CGI less than or equal to two (48), CGI-I less than or equal to 2 and 25% reduction in YBOCS (48). However, the studies mentioned so far do not show a significant advantage over SSRIs. Its efficacy is probably comparable to that of clomipramine, with a side-effect profile that makes it preferable to the latter (46).

## 4. Add-on treatments

### 4.1. Antidepressant combination

Although supported by little evidence, the add-on of clomipramine in combination with SSRIs is considered by the APA Practice Guidelines (8). A 2008 trial, including 20 patients who had failed to respond to at least two trials with a SSRI and who were taking clomipramine at different doses, showed a significant response in 50% of the sample with citalopram as an add-on therapy after 1 month of treatment (49). Another work on 14 patients showed that the add-on of sertraline to clomipramine is preferable to a dose increase of clomipramine as monotherapy in case of resistance (50). A report on four cases also showed that the combination of clomipramine and fluoxetine can be effective even in cases where the individual molecules have not produced any benefit in patients (51).

### 4.2. Antidopaminergic agents

The role of antidopaminergic molecules in the treatment of OCD is suggested by the hypothesis of dopaminergic hyperactivation, with a disruption of the medial prefrontal cortex inhibitory circuit on the amygdala and a subsequent increased activation of anxiety (52, 53). The role of anxiety in the activation of obsessive behavior has been conceptualized from a modeling of complex tasks defined as “structured event complexes” (SECs), with respect to which the orbitofrontal cortex is implicated in reward, the anterior cingulate cortex in error detection, the basal ganglia in influencing the activation threshold of motor and behavioral programmes, while the prefrontal cortex would play the role of storing memories of these SECs. The activation of SECs could be accompanied by anxiety that is progressively alleviated by the performance of tasks, while a deficit in this process may be responsible for many OCD symptoms and have anxiety as its trigger (54). The dopamine D4 (DRD4) variable number of tandem repeats (VNTR) 7R allele polymorphism is significantly associated with OCD (55), and a worsening of obsessive symptoms has been observed in OCD patients taking dopaminergic agonist drugs (56). However, as with serotonin, the evidence is ambiguous. The link between dopaminergic dysfunction and Tourette’s syndrome, as well as for other tic disorders, is more solid, and may in part influence the conception of the pathophysiology of OCD, given the high comorbidity between these conditions.

According to the most recent trials and reviews, the most effective prescriptions are low doses of aripiprazole (1–5 mg/d) and risperidone (0.5–1 mg/d) (57–59). This evidence suggests a possible role for 5HT2A antagonism in the control of OCD symptoms. The addition of an antipsychotic to SSRIs is effective in about a third of patients, especially in the presence of tics, with a number needed to treat of about five (33, 34). A recent meta-analysis including all double-blind, randomized, placebo-controlled trials comparing augmentation of SSRIs with antipsychotics to placebo supplementation in treatment-resistant OCD revealed a clear superiority of haloperidol, aripiprazole and risperidone over placebo, while quetiapine, paliperidone and olanzapine showed no evidence of superiority. Response was defined by a reduction of at least 35% of the YBOCS score. The overall rate of attrition in the group treated with antipsychotics as add-ons ranged from 10 to 25%, attributable in part to adverse effects, the main ones reported being mouth dryness, headache, and increased appetite (36). Clozapine is not recommended, as there is sufficient evidence of its role in a possible worsening of OCD symptoms (60).

### 4.3. Glutamatergic agents

An increased concentration of glutamate, one of the neurotransmitters in the cortico-striato-thalamo-cortical loop, has been detected in the CSF of OCD patients (61, 62), and a specific association between OCD and polymorphisms in genes SAPAP3 and SLC1A1, which code for proteins involved in glutamatergic transmission, has been found in several studies (63–65). The clinical data reported in the following section, however, only offers evidence regarding the use of glutamatergic agents as adjunctive therapies. The etiopathogenetic role of glutamatergic disruption therefore remains to be explored, to clarify whether it is a sufficient cause or rather an added element in the determination of a polyfactorial clinical picture.

The efficacy of memantine, a NMDA receptor antagonist which regulates the effects of pathologically elevated glutamate levels, in the treatment of OCD has been studied in a randomized trial of 42 patients treated with memantine versus placebo as an add-on to fluvoxamine for 8 weeks. At the end of the study, 89% of patients on memantine met the criteria for remission, defined as YBOCS score less than or equal to 16 compared with 32% in the placebo group (66). A recent meta-analysis confirmed that patients receiving memantine were 3.61 times more likely to respond to treatment than those receiving placebo, with a response threshold set at a 35% reduction in the YBOCS score. The average reduction was of 12 points on the YBOCS compared to the scores before the add-on for the treatment group. The most common memantine-related side effects were headache, drowsiness, confusion and dizziness, usually of moderate and transient magnitude. No statistically significant differences in terms of adverse events and dropouts were reported comparing the memantine-treated group and the placebo group (67). Since a significant effect was observed in trials of memantine as add-on therapy after 12 weeks, this period is the minimum recommended for evaluating the appropriateness of this treatment strategy (68).

A recent trial compared amantadine, another glutamatergic agent, versus placebo as add-on therapy to fluvoxamine in a randomized sample of 100 patients for 12 weeks. At the end of the study, the amantadine-treated group had a significant reduction in YBOCS on the total score and on the subscale for obsessive symptoms. No significant differences were observed in the reduction of the subscale for compulsive symptoms. The two groups had no significant differences in adverse effects. The considerations made so far, in the light of this evidence, suggest a potential role for amantadine in the treatment algorithm for OCD (69).

Ketamine is a NMDA receptor antagonist as well as a non-selective agent targeting the opioid, cholinergic and monoamine systems, all of which may contribute to its efficacy in OCD (69–71). It is used in off-label clinical practice as an augmentation strategy when the better-proven approaches have failed (71–73). Most trials indicate a rapid but short-lasting effect (days to weeks), with responses varying from full remission to no benefit (74, 75).

Lamotrigine is an antiepileptic drug used in the maintenance treatment of bipolar disorder. In view of its inhibitory action on AMPA glutamatergic receptors, its possible role as an adjunctive therapy in OCD has been investigated in a few studies and case reports (75–78) all indicating that lamotrigine may be an effective and safe therapeutic option as an add-on to SSRI treatment.

Topiramate, another AMPA antagonist, showed controversial results as both improvements (79) and worsening (80) of symptoms were observed.

## 5. OCD resistant vs. OCD refractory

A patient meets the criteria for OCD resistant when he or she has a reduction inferior to 25% at the YBOCS despite a trial of at least 12 weeks at the highest tolerated dose of SSRIs or clomipramine, in combination with at least 30 h of CBT. Refractory OCD is defined as a non-response after 3–6 months of at least three antidepressants (including clomipramine), and at least two add-on trial with atypical antipsychotics (81). However, these operational definitions are not unequivocal in the literature, and there are those who reserve the category of refractory for those patients who show no benefit or even worsen with the proposed treatment (82). Even in cases where adequate treatment is offered to the patient, a 10% persistence of severe disability due to OCD can be observed (83). For these patients, one therapeutic option may be the addition of glutamatergic agents, according to the potential and limitations just described. As an additional criterion for the transition to interventional therapy, the use of another CBT trial with a second independent therapist is indicated as a consensual criterion. Regarding interventional psychiatry, the strongest evidence is currently available for the use of DBS, while preliminary data encourage the investigation of other alternatives.

### 5.1. DBS

Deep brain stimulation (DBS) is a neuromodulation technique whose application in OCD is based on a well-documented efficacy (84). A systematic review showed that, with regard to the target, there were no significant differences between the anterior limb of the internal capsule (ALIC) and the subthalamic nucleus (STN), and that up to 60% of operated patients had a reduction of at least 35% at YBOCS (85). The authors do not systematically report the inclusion criteria for all patients presented in the meta-analysis, but state that DBS is a last-line therapy. The most commonly accepted criteria for the indication of DBS in OCD patients are as follows: non-response (response being defined as at least 35% reduction in YBOCS) to two courses of SSRI treatment at the maximum dosage for at least 12 weeks; one course of clomipramine treatment at the maximum dose for at least 12 weeks; one add-on therapy with a second-generation antipsychotic for at least 8 weeks; one course of CBT, a Y-BOCS score of at least 28 points; a GAF score of less than 45 points; OCD duration of at least 5 years (79). This should be completed by the findings of a 2015 survey of 18 patients investigating the overall impact of DBS in quality of life (86). Both YBOCS responders and non-responders reported an improvement in their condition, while also reporting an improvement in their self-perception and emerging difficulties in the social sphere. These data are comparable with what has been learned about Parkinson’s patients who benefited from STN DBS (87). More recently, new targets have been proposed, such as ventral capsule/ventral striatum (VC/VS), nucleus accumbens (NAcc), anteromedial subthalamic nucleus (amSTN), or inferior thalamic peduncle (ITP) (84). The preliminary evidences of efficacy leaves open the prospect of an individual approach based on the identification of the different dimensions contributing to the heterogeneity of OCD (88). To date, the Congress of Neurological Surgeons considers the following evidence-based recommendations: the use of bilateral subthalamic nucleus DBS, combined with optimal pharmacotherapy, is recommended on a level I evidence basis. The use of bilateral nucleus accumbens or bed nucleus of stria terminalis for refractory pathology is on a type II level of evidence. These indications are the result of a systematic review conducted by the Guidelines Task Force in 2020, considering an updated literature up to 2019 (89).

Although DBS may be a viable treatment option to consider in resistant OCD, a recent Swedish survey revealed that only 29% of OCD patients are aware of its existence, that all psychotherapists surveyed estimate that their patients do not meet the criteria for an intervention, and that although psychiatrists believe 98% of the time that they have patients potentially eligible for DBS, they doubt their ability to identify them (90).

Added to this is the difficulty of insurance coverage: in the US, only 50 per cent of potential DBS recipients receive treatment, and less than 40 per cent receive coverage from their insurance company (91).

A 2022 article on the DBS access crisis identifies the lack of insurance and lack of knowledge on the part of mental health professionals as the cause of this The authors point out that this is in contrast to the mental-health parity laws enacted in 2008 (92).

A recent review, which included 40 articles and covers the last 20 years of DBS practice in OCD patients, reports the main adverse effects associated with this therapy. These can be divided into three groups: adverse effects due to surgical or hardware-related complications, stimulation-induced side effects and other types of side effects which will be listed briefly below. Electrode malpositioning or intracranial infection (which affects between 1 and 15% of Parkinson’s DBS procedures overall) are the main causes of device removal and re-implantation. Intracranial bleeding is a serious side effect that can reach rates of between 4.8 and 7.7%. Epileptic seizures, regardless of the site of stimulation, have been occasionally described in the 5 years following surgery. These have malpositioning, cranial infections, unstable somatic pathologies and abrupt changes in parameters as risk factors. The most frequent stimulation-related side effect is hypomania, although this usually resolves after adjustment of the stimulation parameters. Other adverse effects related to stimulation include weight gain, sleep disturbances, subjective memory complaints and increased anxiety. The increased risk of suicide remains controversial as this could be attributable to previous pathology or disappointment at the lack of response to the device implantation (93).

### 5.2. Other interventional techniques

#### 5.2.1. rTMS

A review of 2011 (94) reports 10 studies on repetitive transcranial magnetic stimulation (rTMS) targeting dorsolateral prefrontal cortex (DLPFC), orbitofrontal cortex (OFC) and supplementary motor area (SMA), stating that it only demonstrated acute efficacy, with no significant difference with sham treatment. The most frequently reported adverse event in rTMS studies is headache, while there are anecdotal case reports on the occurrence of seizures and psychotic symptoms. In the meta-analysis under discussion, none of the side effects persisted for more than 4 weeks after the end of stimulation and no serious adverse events such as seizures and memory problems or cognitive problems occurred.

More recently, a multicenter study showed that bilateral low frequency rTMS targeting SMA significantly reduced obsessive symptoms compared to sham, with a sustained effect at 6 weeks follow-up (95). Another study showed the superiority of a 1 Hz stimulation of DLPFC over a similar 10 Hz stimulation and sham (96). Specific coils have received FDA approval for the treatment of OCD: H7 was cleared in 2018, based on evidence showing that its use in one study led to a 30% reduction in YBOCS in 38% of treated patients, compared to 11% in sham conditions (97). In 2020, the COOl D-B80 coil also received approval. According to the Clinical TMS society, the use of FDA-approved coils for OCD is recommended in case of resistance after two indicated therapies (two medications or one medication plus psychotherapy) that have been conducted for at least 8 weeks, or in case of drug intolerance after at least two trials. TMS is considered a viable alternative to relatively risky second- and third-line drug trials, such as antipsychotics, opioids, benzodiazepines and glutamatergic agents (98).

This evidence suggests that planning rTMS therapy before giving an indication for DBS is certainly desirable, considering the risks and benefits.

#### 5.2.2. tDCS

A 2021 meta-analysis on the use of tDCS in psychiatric and neurological disorders found that a Pubmed search for the two keywords “tDCS” and “OCD” yielded a result of only eight entries. Due to the scarcity of trials, the authors exceptionally included Class IV studies in their analysis of this disorder, without excluding those involving a pediatric population (99). In this context, the authors make a recommendation of anodal pre-SMA tDCS as possibly effective in improving OCD (Level C). This is based on a class II trial (100) in which non-responders were enrolled in a subsequent open-label phase, achieving a noticeable improvement in symptom intensity despite not being able to be considered as responders. Further research is needed in this area, given these encouraging preliminary data on a technique characterized by safety and minimal invasiveness.

According to a review taking into account 567 tDCS sessions, the adverse events reported were moderate fatigue (35.3%), tingling (70.6%), slight itching at the electrode placement site (30.4%), headache (11.8%), nausea (2.9%), and insomnia (0.98%) (101). Despite its excellent safety profile, the data on the potential efficacy of tDCS in OCD do not currently justify systematically proposing this therapy in resistant or refractory cases.

## 6. Discussion

Less than 10% of OCD patients are currently receiving evidence-based therapy (10). The prospect of improving the offer for these patients, suffering from a highly debilitating condition lies in the adoption of common and scientifically validated practices on the one hand, and also in focusing research efforts in the directions offered by interventional psychiatry, and specifically DBS.

**FIGURE 1:**
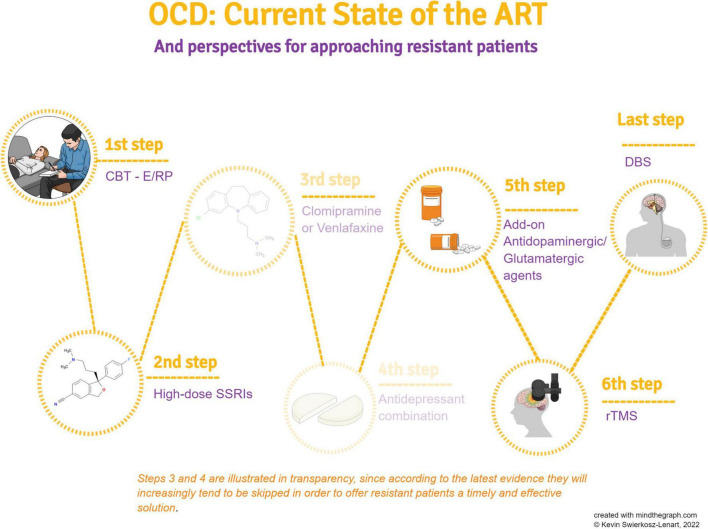
A schematic summary of the various treatment steps according to the best evidence in the literature.

The scrupulous adoption of diagnostic and assessment criteria, followed by the adoption of treatment guidelines, allows reliable identification of resistant cases, which are potential beneficiaries of therapeutic approaches under research investigation. Given the impact of the disease on the patient’s quality of life, there is an increasing need to bring clinicians and researchers together to propose guidelines that integrate treatment options at the different stages of the algorithm. A graphic summary of the evidence discussed in this article is presented in Figure 1. There is a need to propose the transfer of “experimental” paradigms to the clinic, without formally demanding a high level of evidence-basis in cases of resistance, but rather focusing on sufficient data to allow clinicians to make proposals according to the clinical presentation. Clinics and research are moving together in a direction of local groups developing empirical strategies supported by a reasonable foundation. It is important for this work to continue because it is from there that solid evidence will come to produce future guidelines, capable of integrating the most recent pharmacological and technical acquisitions.

## Author contributions

JD, JE, A-HC, JB, FR, JFB, BD, M-TC, BPM, AG, and LM contributed equally, either by writing entire paragraphs in their specific field of expertise, or by providing bibliographical references, and correcting and expanding information in order to offer the reader the best state-of-the-art in the article’s field of investigation. All authors contributed to the article and approved the submitted version.
